# The role of *Dmnt1* during spermatogenesis of the insect *Oncopeltus fasciatus*

**DOI:** 10.1186/s13072-023-00496-5

**Published:** 2023-07-01

**Authors:** Christopher B. Cunningham, Emily A. Shelby, Elizabeth C. McKinney, Robert J. Schmitz, Allen J. Moore, Patricia J. Moore

**Affiliations:** 1 Dept. of Entomology, University of Georgia, Athens, GA USA; 2 Dept. of Genetics, University of Georgia, Athens, GA USA

**Keywords:** DNA methylation, Epigenetics, Germ Cell Production, Hemiptera, Reproduction

## Abstract

**Background:**

The function of DNA methyltransferase genes of insects is a puzzle, because an association between gene expression and methylation is not universal for insects. If the genes normally involved in cytosine methylation are not influencing gene expression, what might be their role? We previously demonstrated that gametogenesis of *Oncopeltus fasciatus* is interrupted at meiosis following knockdown of *DNA methyltransferase 1* (*Dnmt1*) and this is unrelated to changes in levels of cytosine methylation. Here, using transcriptomics, we tested the hypothesis that *Dmnt1* is a part of the meiotic gene pathway. Testes, which almost exclusively contain gametes at varying stages of development, were sampled at 7 days and 14 days following knockdown of *Dmnt1* using RNAi.

**Results:**

Using microscopy, we found actively dividing spermatocysts were reduced at both timepoints. However, as with other studies, we saw *Dnmt1* knockdown resulted in condensed nuclei after mitosis–meiosis transition, and then cellular arrest. We found limited support for a functional role for *Dnmt1* in our predicted cell cycle and meiotic pathways. An examination of a priori Gene Ontology terms showed no enrichment for meiosis. We then used the full data set to reveal further candidate pathways influenced by *Dnmt1* for further hypotheses. Very few genes were differentially expressed at 7 days, but nearly half of all transcribed genes were differentially expressed at 14 days. We found no strong candidate pathways for how *Dnmt1* knockdown was achieving its effect through Gene Ontology term overrepresentation analysis.

**Conclusions:**

We, therefore, suggest that *Dmnt1* plays a role in chromosome dynamics based on our observations of condensed nuclei and cellular arrest with no specific molecular pathways disrupted.

**Supplementary Information:**

The online version contains supplementary material available at 10.1186/s13072-023-00496-5.

## Background

Cytosine methylation of insect DNA remains an enigma. The addition of a methyl group to the nucleotide cytosine is important to cell function and survival of vertebrates and plants [27, 42]. For many domains of life, cytosine methylation is an epigenetic mechanism that negatively regulates gene expression [42]. The cytosine methylation of insects is different. Cytosine methylation is highest within exons and not gene promoter regions [14, 17] Gene body methylation levels across insects is associated with highly and broadly expressed genes [13, 17, 28, 49]. However, differences of cytosine methylation are rarely directly associated with difference of gene expression [13, 34], but might be associated with reduced variation of expression [36]. The function of methylation for insects appears to be lineage specific [32, 47] and direct functional manipulation of *Dmnt1* implicates a role in reproduction independent of cytosine methylation [4, 8, 22, 48]. In addition, cytosine methylation itself and its machinery are variably conserved across most insect lineages [7, 13, 14, 39], although most insects have copies of methyltransferases [7]. Indeed, this variation can be extreme. Some insects, such as *Drosophila melanogaster*, lack DNA methyltransferases and others, such as *Tribolium castaneum*, have *Dmnt1* but lack cytosine methylation in their genome [7, 39]. Thus, the function of *Dnmt1* of insects beyond its maintenance of cytosine methylation after DNA replication [1], and why its presence is variable, remains unclear.

One recently discovered role for the cytosine methylation machinery of insects is during gamete and embryo formation [3, 4, 8, 16, 22, 43, 48, 51]. In addition, for the large milkweed bug *Oncopeltus fasciatus*, *Dmnt1* has been identified as critical during meiosis [4, 48]. This association with gamete formation may provide an insight into the variability of DNA methyltransferases of insects. Mechanisms of insect gamete formation is highly diverse [15, 20], and if *Dnmt1* is related to meiosis during gametogenesis this may explain the diversity we see of this gene for insects. To test this idea, here we investigated if *Dnmt1* is associated with meiotic pathways by investigating gene expression in a priori defined meiosis-related genes. We also use transcriptomics to compare *Dnmt1* gene expression in knockdown vs control insects, and examine associations between *Dnmt1* expression and other genes and pathways.

The large milkweed bug *O. fasciatus* has relatively high levels of cytosine methylation, as well as functional single copies of *Dmnt1*, *Dmnt2*, and *Dmnt3* [8]. Our previous studies investigated *Dnmt1* during gamete formation of both ovaries and testes given these are rapidly dividing cells and *Dnmt1* replicates cytosine methylation after a cellular division [42]. When adult female *O. fasciatus* are injected with *Dnmt1* RNA interference (RNAi), they fail to produce viable eggs and gamete production stops [8]. When injected during a juvenile stage prior to formation of primary oocytes via meiosis in the developing ovary, *Dnmt1* knockdown completely ablates gamete formation but the somatic ovaries develop normally [4]. When *Dnmt1* is knocked down during a pre-meiosis stage of juvenile testis development, resulting in a large reduction of cytosine methylation within the testes, males emerge as adults with significantly smaller testes that have fewer developing sperm [48]. Similar to females, when injected as adults, spermatogenesis is blocked and no further sperm develop resulting in reduced fertility once sperm that had been in the process of development at the time of treatment have been used up [48]. These results indicate that the reduction of *Dnmt1* specifically affects production of gametes rather than gamete maturation. This led us to hypothesize that *Dnmt1* influences gametogenesis at meiosis.

With this study, we used RNA-seq on RNAi and control injected *O. fasciatus* males to test the hypothesis that *Dnmt1* influences spermatogenesis by influencing the expression of meiosis genes. In females, this reduction of fecundity is associated with the differential gene expression of several hundred genes [8]. Like other insects, this differential gene expression is not associated with differential methylation [8, 13]. However, if *Dnmt1* specifically affects the primary oocytes, which represent a small proportion of the cells within the adult ovary, it may be that any specific signatures of differential gene expression might be masked by the large number of unaffected somatic cells. The situation in the testis is very different. Each testis tubule is comprised of spermatocysts containing sperm at varying stages of development, and most cells within the testis are spermatogonia, spermatocytes, or developing spermatids. Given that the underlying phenotype of males and females appears to be similar, an impediment to the transition from germ cell (the diploid oogonia or spermatogonia) to gametes, we compared the gene expression pattern of testes following *Dnmt1* knockdown using RNAi. For this species, targeting *Dnmt1* with RNAi is very robust producing similar phenotypes and reductions of cytosine methylation for both males and females using multiple different constructs and controls [8, 48], including the injection timing and tissue used here. To capture any early events as opposed to more global changes that might result from downstream impacts of impeded development not directly related to the knockdown of *Dnmt1*, we sampled testes at 7-day and 14-day post-injection, which are earlier than the 21-day post-injection samples taken in the Washington et al. [48], where testes have significantly reduced numbers of developing spermatocytes and a highly disrupted structure. With these earlier sampling points, we aimed to capture the gene expression changes that were leading to the developmental arrest of spermatogenesis, presumably at the transition between diploid spermatogonia and haploid spermatocytes. We also tested our broad hypothesis of post-mitotic-specific arrest using a priori candidate genes representing different reproductive, molecular pathways and used all expressed gene to test if meiosis GO terms were enriched.

## Results

### *Dnmt1* RNAi knockdown

*Dnmt1* was effectively knocked down in experimental males treated with ds-*Dnmt1* RNA. Using qRT-PCR, we confirmed *Dnmt1* was downregulated in the ds-*Dnmt1* samples (7 days: mean fold expression difference = -0.677, *t*_26_ = 7.250, *P* = 8.609e-8; 14 days: mean fold expression difference = -0.679, *t*_26_ = 4.288, *P* = 0.00011). For both data sets (7 days, 14 days) *Dnmt1* was statistically significantly downregulated in *Dnmt1* RNAi treatment (Table 1).

**Table 1:** a priori candidate genes tested for differences between 7- and 14-day Control and *Dnmt1* knockdown treatments

Gene Name	Gene ID	Pathway	7 days			14 days		
			Standardized expression	Fold change	FDR-corrected P	Standardized expression	Fold change	FDR-corrected P
*Dnmt1*	OFAS015351, OFAS018396	Cytosine methylation	**7.9**	**− 0.185**	**1.20E-03**	**6.3**	**− 8.72E-01**	**2.20E-16**
*Wnt*-like	OFAS019215	Cell fate and proliferation	NA	NA	NA	NA	NA	NA
*Wnt*-like	OFAS025063	Cell fate and proliferation	7.1	1.25E-06	0.994	5.5	0.142	0.621
*Frizzled*-like	OFAS012753	Cell fate and proliferation	7.3	− 4.82E-06	0.037	5.7	− 0.302	0.226
*Frizzled*-like	OFAS013300	Cell fate and proliferation	9.5	− 7.16E-06	0.245	7.8	0.047	0.766
*Frizzled*-like	OFAS013301	Cell fate and proliferation	7.8	1.03E-06	0.955	6.3	− 0.036	0.875
*Vasa*	OFAS025106	Germ cell Development	10.7	− 3.68E-06	0.663	**8.6**	− **0.622**	**0.013**
*CYCB3*	OFAS004926	Cell cycle control	7.9	1.29E-06	0.618	5.8	− 0.337	0.268
*CDC20*	OFAS011085	Cell cycle control	12.9	1.07E-05	0.853	**10.7**	− **0.743**	**7.47E-05**
*CDC25*-like	OFAS004022	Cell cycle control	9.0	− 4.22E-05	0.336	**7.0**	− **0.575**	**0.004**
*CDC25*-like	OFAS004025	Cell cycle control	7.1	− 5.29E-05	0.938	5.5	0.027	0.925
*CYCD2*	OFAS016512	Cell cycle control	NA	NA	NA	NA	NA	NA
*CYCD3*	OFAS016513	Cell cycle control	6.4	2.72E-06	0.149	5.2	0.384	0.178
*SMC3*-like	OFAS014329	Chromosomal Structure Maintenance	7.9	1.43E-05	0.111	5.9	− 0.204	0.475
*SMC3*-like	OFAS014330	Chromosomal Structure Maintenance	6.7	− 7.77E-05	0.971	5.1	− 0.549	0.092
*Boule*	OFAS007982	meiotic G2/M transition	12.3	− 1.98E-06	0.121	**10.2**	− **0.573**	**0.013**
*SPO11*	OFAS017698	Meiotic Recombination	9.2	8.39E-07	0.777	7.1	− 0.453	0.052
*MSH5*	OFAS011763	Meiotic Recombination	6.5	1.18E-05	0.319	**5.2**	**1.916**	**0.001**
*MND1*	OFAS003294	Meiotic Recombination	9.6	− 9.14E-05	0.220	7.5	− 0.258	0.157
*HOP2*	OFAS000825	Meiotic Recombination	8.3	2.24E-05	0.032	6.7	0.346	0.088

*Dnmt1* is a split annotation but is a single gene [8]. Values were standardized using the vst function of DESeq2. Bolded values are statistically significant after Bonferroni–Hockberg FDR Correction. NA expression was not detected. *P* values were combined with Fisher's method

### Microscopy—Effect of *Dnmt1* RNAi knockdown

**Fig. 1:**
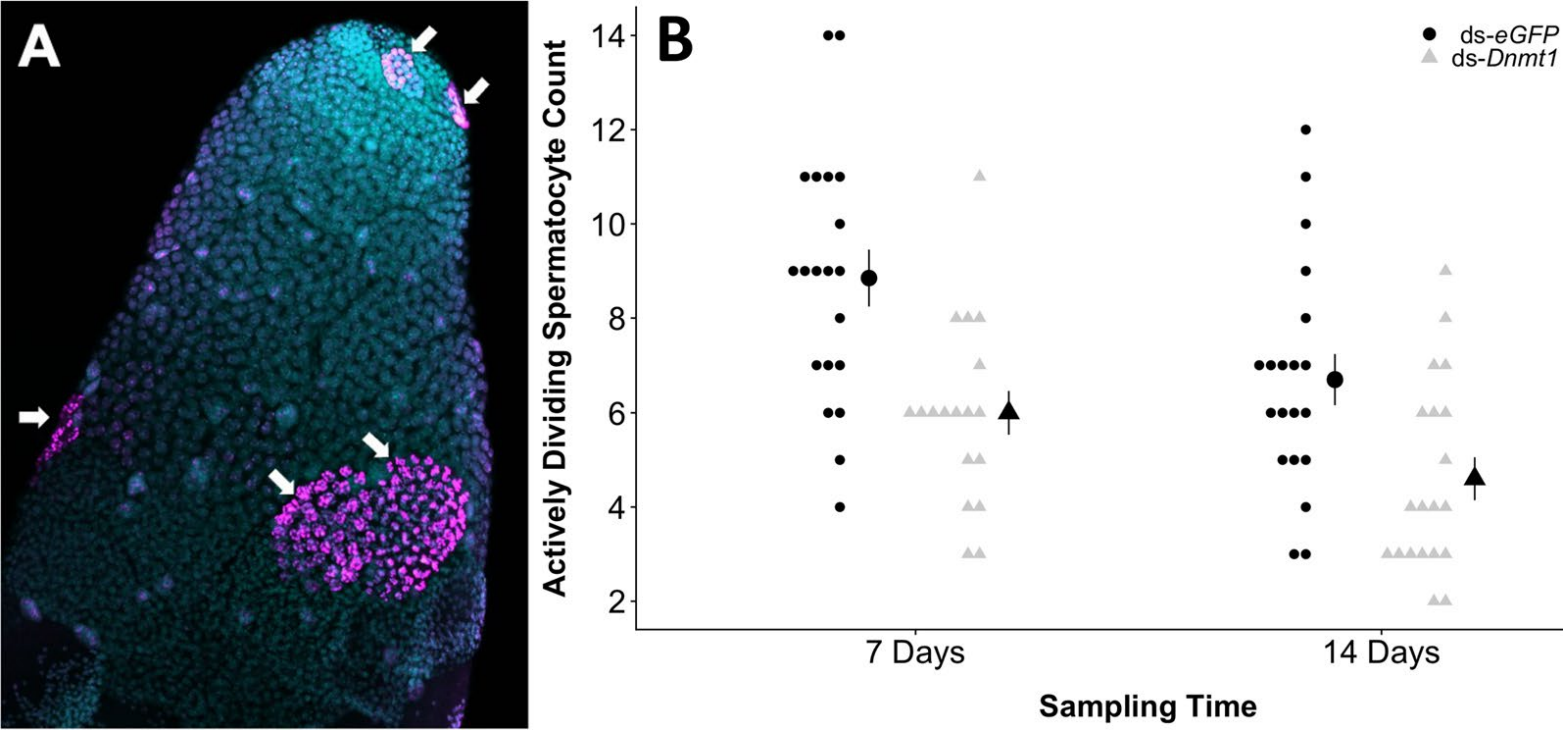
Male gamete formation is highly perturbed following knockdown of *Dnmt1* revealed by testes tubules stained with anti-phosphohistone H3 antibody. **A** Representative image of an *eGFP* testis tubule stained with anti-pHH3 antibody and DAPI. The spermatocysts that were in the act of dividing at the time of dissection are labeled with magenta (arrows). 20X magnification. **B** There were fewer dividing cells in *Dnmt1* knockdown males at both sampling times post-RNAi treatment compared to controls. Each individual symbol represents a biological replicate (*n* = 20 per treatment). Summaries as larger symbols are presented as mean ± standard error

Knockdown of *Dnmt1* had a measurable phenotype in males sampled at both 7- and 14-day post-injection. Knockdown of *Dnmt1* reduced the number of actively dividing spermatocysts (Fig. 1A). The number of spermatocysts that were positively stained with anti-pHH3 antibody, which indicates cell undergoing mitotic or meiotic division, was reduced in males at both 7-day post-knockdown (mean difference = − 3.45, *t*_38_ = 4.098, *P* = 0.0001; Fig. 1B) and 14-day post-knockdown (mean difference = − 2.1, *t*_38_ = 2.978, *P* = 0.0025; Fig. 1B).

### a priori candidate genes

There were no differentially expressed a priori candidate genes at 7 days (Table 1). Six of seventeen were at 14 days (Table 1). However, they did not concentrate around a particular process. Three of six cell cycle control genes were differentially expressed, while two of five meiotic genes were differentially expressed.

### a priori GO term enrichment

Using every expressed gene for analysis, none of the GO terms were enriched at 7 or 14 days after *Dnmt1* knockdown (Table 2). In fact, contrary to our expectation, all of the five GO terms that we made predictions for were absent from the top of the list of genes rank-ordered by difference of expression at 7 days (i.e., they were statistically preferentially found at the bottom of the list of genes rank-ordered by their expression differences), while none were overrepresented at either the top or bottom of the rank-ordered list at 14 days (Table 2). The necrotic and apoptotic GO terms were not enriched at either sampling point.

**Table 2:** a priori GO term enrichment at 7 days and 14 days

	Pathway	GO Term	Number of annotated genes	NES	*P* value	FDR-adjusted *P* value
7 days	Cell Cycle	GO:0007049	1045	**− 3.224**	**5.34E-11**	**3.74E-10**
	Gametogenesis	GO:0007276	1275	**− 3.026**	**1.92E-10**	**6.73E-10**
	Mitotic Cell Cycle	GO:0000278	738	**− 2.813**	**3.47E-08**	**8.10E-08**
	Meiotic Cell Cycle	GO:0051321	329	**− 2.519**	**2.93E-05**	**4.11E-05**
	Spermatogenesis	GO:0007283	493	**− 2.371**	**2.25E-05**	**3.93E-05**
	Apoptotic Process	GO:0006915	426	**− **0.973	4.95E-01	5.77E-01
	Necrotic Cell Death	GO:0070265	21	**− **0.634	9.20E-01	9.20E-01
14 days	Cell Cycle	GO:0007049	1031	0.839	1	1
	Gametogenesis	GO:0007276	732	0.884	1	1
	Mitotic Cell Cycle	GO:0000278	324	0.846	1	1
	Meiotic Cell Cycle	GO:0051321	1256	0.872	1	1
	Spermatogenesis	GO:0007283	486	0.853	1	1
	Apoptotic Process	GO:0006915	422	0.939	0.938	1
	Necrotic Cell Death	GO:0070265	20	0.891	0.755	1

This aligns with a general, global response to the loss of *Dnmt1* not heavily, preferentially targeting one or a few pathways. NES normalized enrichment score. Statistically significant values are bolded

### Differential gene expression

**Fig. 2:**
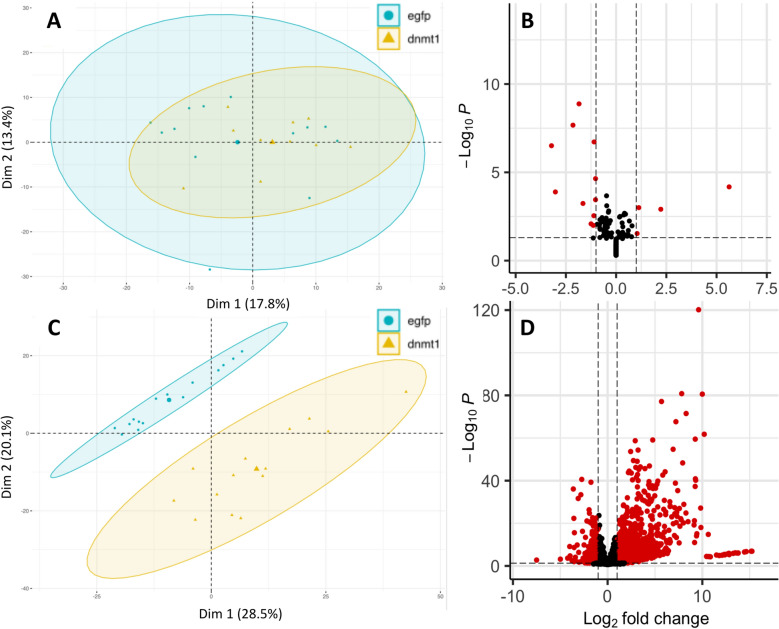
Gene expression knockdown of *Dnmt1* leads to increasing differential gene expression as testes initiate and progress through gamete formation after treatment. Here, samples are visualized with a Principal Component Analysis visualization. Each sample is represented by a smaller symbol corresponding to its treatment, while the larger symbol represents the centroid of the treatment. Differential gene expression is presented with a Volcano plot; each dot representing a gene. **A** Samples 7-day post-treatment are highly overlapping and not differentiated by many gene expression differences. **B** Very few genes were statistically significantly differentially expressed (represented as red dots). **C** Samples 14-day post-treatment are highly non-overlapping and differentiated by many gene expression differences. **D** Many genes are statistically significantly differentially expressed at 14 days (~ 45% of expressed genes are different between the treatments)

We investigated gene expression at two sampling points—7 and 14 days—post-injection to identify the genes that are differentially expressed after knockdown of *Dnmt1*, which leads to the systematic depletion of spermatocysts among adult *O. fasciatus* testes (number of biological replicates: 13 7-day *eGFP*, 10 7-day *dnmt1*, 15 14-day *eGFP*, and 14 14-day *Dnmt1*). The individual treatments clustered together well at both sampling points (Fig. 2). The treatments are not differentiated at 7 days (Fig. 2A; Additional file 3), are but highly differentiated at 14 days (Fig. 2C; Additional file 4). There were 74 genes that were differentially expressed between the control treatment and the ds-*Dnmt1* treated samples at 7-day post-injection (23 up-regulated; 51 down-regulated; Fig. 2B). There were 6,746 genes that were differentially expressed between the control treatment and the ds-*Dnmt1* treated samples at 14-day post-injection (2,761 up-regulated; 3,985 down-regulated; Fig. 2D).

The overlap between the differentially expressed genes found here and those of Bewick et al. [8] using the same treatment against ovaries, was four for the 7-day list and ninety-six for the 14-day list. Both overlaps are statistically significant. The 7-day comparison had more than expected (mean simulated overlap = 1.32 *P* = 0.041) and the 14-day comparison had fewer than expected (mean simulated overlap = 119.24, *P* = 0.0022).

### Gene Ontology (GO) term overrepresentation

Among the differentially expressed genes between *Dnmt1* knockdown and *eGFP* control at 7-day samples, 113 GO terms were statistically significantly overrepresented. The top three Biological Processes terms with more than one gene were purine nucleobase biosynthetic process—GO:0009113, skeletal muscle organ development—GO:0060538, and mitotic sister chromatid cohesion—GO:GO:0007064 (*P* = 0.0014, 0.0016, 0.006, respectively); Molecular Function terms were calcium-dependent phospholipid binding—GO:0005544, cation binding—GO:0043169, and ribose phosphate diphosphokinase activity (*P* = 0.0017, 0.008, 0.0083, respectively); and for Cellular Compartments were nuclear meiotic cohesin complex—GO:0034991, Parkin-FBXW7-Cul1 ubiquitin ligase complex—GO:1,990,452, and integral component of Golgi membrane—GO:0030173 (*P* = 0.0087, 0.0087, 0.0125, respectively). Very few GO terms collapsed under a parent GO term in our semantic similarity analysis trying to understand what broad process was most perturbed with *Dnmt1* knockdown. A complete term list can be found in Additional file 5.

For the 14-day samples, 322 GO terms were statistically significantly overrepresented. The top three Biological Processes terms with more than one gene were response to unfolded protein—GO:006986, double-strand break repair via nonhomologous end joining—GO:006303, and mRNA splice site selection—GO:0006376 (*P* = 0.00011, 0.00016, 0.00019, respectively), Molecular Function terms were nucleoside-triphosphatase activity—GO:0017111, single-stranded DNA binding—GO:0003697, and electron transfer activity—GO:0009055 (*P* = 0.00014, 0.00038, 0.00062, respectively), and for Cellular Compartments were ribonucleoprotein granule—GO:0035770, CCR4-NOT core complex—GO:0030015, sperm flagellum—GO:0036126 (*P* = 0.00015, 0.00025, 0.00025, respectively). Very few GO terms collapsed under a parent GO term in our semantic similarity analysis trying to understand what broad process was most perturbed with *Dnmt1* knockdown. A complete term list can be found in Additional file 6.

For the overlap between the differentially expressed genes found here and those of Bewick et al. [8] for the 14-day samples, we found 168 GO terms. The top three Biological Processes terms with more than one gene were protein K63-linked deubiquitination—GO:0070536, melanocyte differentiation—GO:0030318, and negative phototaxis (*P* = 0.0002, 0.00025, 0.00032, respectively), Molecular Function GO terms were proteasome binding—GO:0070628, alditol:NADP + 1-oxidoreductase activity—GO:0004032, and estradiol 17-beta-dehydrogenase activity—GO:0004303 (*P* = 0.00012, 0.00029, 0.000221, respectively), and for Cellular Compartments were molybdopterin synthase complex—GO:0042629, apical cortex—GO:0045179, and outer dynein arm—GO:0036157 (*P* = 0.00017, 0.00691, 0.00072, respectively). Very few GO terms collapsed under a parent GO term in our semantic similarity analysis. A complete term list can be found in Additional file 7.

## Discussion

Producing gametes is achieved by a surprisingly diverse set of species- and lineage-specific set of mechanisms and we suggested that *Dnmt1* may be part of this diversity. Our previous work with *O. fasciatus* shows that the genes for cytosine methylation are needed for successful gamete production [4, 8, 48] but the mechanistic underpinnings of this effect are unclear. Here, we reduced the gene expression of *Dnmt1* of sexually mature *O. fasciatus* males. Given the phenotype observed here and previous studies, we predicted meiosis, and its molecular pathways would be highly perturbed, giving rise to a loss of meiotic progression and a cessation of spermatocyte production observed. Yet our candidate gene screen looking for differential expression of a priori predicted genes did not reveal an obvious molecular pathway. Both the collection of cell cycle and meiotic genes contained differentially expressed genes, but it was not the majority of either group; 3/6 for cell cycle and 2/5 for meiotic genes. These results align with the observation that cells appear to arrest at the meiotic stage, do not progress through further cell division which would require completed cell cycles, and that *Dnmt1* might have a cytosine methylation independent function during reproduction.

As with the candidate gene analysis, our hypothesis received little support from our GO term enrichment analysis. None of the seven GO terms that we tested were enriched at 7 days (i.e., the genes with those GO terms were not preferentially found at the beginning of the rank-ordered gene list). In fact, the GO term searched were preferentially found at the end of the gene list at the 7-day mark, except for necrotic and apoptotic processes which did not have statistically significantly pattern (i.e., were among genes that showed the smallest differences of expression between the control and *Dnmt1* knockdown samples). Like our candidate gene results, this suggests that few pathways were perturbed at this sampling point and there is no evidence that the cells are aborting themselves. At 14 days, none of the GO terms were enriched nor were they preferentially found at the end of the rank-ordered gene list. This means that these genes collectively increased their rank within the rank-ordered gene lists based on expression differences from 7 days to 14 days, but not at a rate that made them statistically enriched at the top of the 14-day gene list. The necrotic and apoptotic GO terms were not enriched at either timepoint suggesting that cells were not actively promoting either of these processes at either sampling point. Taken together, we suggest this supports the hypothesis that the gene expression difference at 14 days is a global response to *Dnmt1* knockdown and is not highly targeted to any specific set of molecular pathways. In general, the cells do not have a detectable signal that they are undergoing any process to actively remove themselves from the cell population.

Given the lack of support for our hypothesis of a role for *Dmnt1* in a meiosis pathway, we used the transcriptome to generate post hoc hypotheses. We found few gene expression difference at 7 days, but a large amount at 14 days post-treatment (~ 45% of expressed genes are differentially expressed). There was no clear consensus among the overrepresented GO terms to suggest how this cellular arrest is being achieved. All of the top five GO terms produced by the semantic similarity reduction for any three of the categories for both days were single standalone terms. The overlap between Bewick et al. [8] and our samples also did not produce any strong candidate pathways for *Dnmt1*’s influence after reduction. These pieces of evidence suggest that gametes are proceeding along a developmental pathway until *Dnmt1* is needed, and when *Dnmt1* is not present due to gene knockdown, the cell has a massive, pervasive, and non-specific disruption of its transcription environment. It appears that the differential gene expression seen is being driven by a healthy vs arrested cell contrast and that the effect of a reduction of *Dnmt1* expression is a general, global response tied to chromatin condensation, not a set of specific genomic loci that are being targeted. Our RNA-seq results suggest that testes with reduced *Dnmt1* expression have an increasingly degradation of their transcriptional environment. The 74 differentially expressed genes of the 7-day samples did not contain many transcription factors or developmental pathway genes as we expected. At 14 days, nearly half of all the expressed genes are differentially expressed—6794 of 14,984 genes (45.3%). Within ovaries treated in the same way and sampled at 10-day post-treatment, there were an intermediate amount of 236 differentially expressed genes [8]. There was little overlap from our 7 days differentially expressed gene list with the ovary samples and fewer than statistically expected with the 14-day list.

We propose that *Dnmt1*’s action during gametogenesis influences chromosomal dynamics and nuclei condensation, based on multiple and repeatedly seen lines of evidence in our experiments. Gene expression knockdown of *Dnmt1* does lead to reduced cytosine methylation [4, 8, 48]. However, cytosine methylation differences are not directly causal for differential gene expression for *O. fasciatus* [8], or for insects generally [13]. This suggests that *Dnmt1* actions to produce this phenotype are not driven by differences of cytosine methylation. When gene expression of *Dnmt1* is reduced hundreds to thousands of gene are differentially expressed for both male and female reproductive tissues [8], this study). We posit that the differential gene expression seen between *Dnmt1* gene expression knockdown and control samples is attributable to comparing samples, where one set is naturally progressing through gamete formation, while the other is in a highly arrested state. It does not appear that this arrest is an active process that the cells are undergoing, but rather is a function of the cell not being able to progress. This aligns with there being no signal from the GO term enrichment the cells are undergoing any cellular death process; necrotic or apoptotic; and that the cell looks healthy at a gross anatomical level. Even though differences of methylation of individual genomic loci do not lead to differences of gene expression between treatments, high cytosine methylation is associated with high gene expression between different loci [17, 18, 28, 49]. This high methylation–high expression association is particular to insects in contrast to plants, fungi, and vertebrates for which high cytosine methylation is often associated with heterochromatin [42]. Highly condensed nuclei are seen for testis tubules with gene expression knockdown of *Dnmt1* [48]. This effect is most pronounced at the region, where spermatocysts transition from spermatogonia to spermatocytes through meiosis [48]. When *Dnmt1* gene expression is reduced by RNAi, it also leads to a reduction of cytosine methylation of somatic tissues [4]. However, this reduction does not impact life span, suggesting that the lack of cytosine methylation within somatic tissues does not have a strong impact on the somatic cells of the organism [4]. This also points to a difference between somatic and reproductive cells which aligns with the presence and absence of meiosis. What remains to be directly explained by this model is why reduced *Dnmt1* gene expression knockdown or a lack of cytosine methylation is associated with highly condensed chromatin (or conversely an inability to de-condense chromatin) as gametogenesis progresses from earlier to later stages.

Careful molecular natural history has exposed much variation across insects for the level of gene body methylation [7, 39] and that differences of methylation are not casually associated with differences of gene expression [13]. A role for *Dmnt1* in chromosome dynamics addresses why its actions appear crucial yet independent function during reproduction, why cytosine methylation has persisted for insects, and why it is rarely associated with gene expression across the taxon. Even with this suggestion, it remains possible that cytosine methylation could directly influence gene expression in a subset of cells even if it does not systemically. This mechanism of action would explain why a reduction of *Dnmt1* expression (1) leads to a highly perturbed transcriptional environment but why cytosine methylation is not associated with gene expression directly, (2) why *Dnmt1* RNAi tissues have high condensed nuclei at the boundary, where spermatocysts usually enter the first meiotic division, and (3) why *Dnmt1* has a specific and critical role during meiosis that likely is not driven by difference of DNA methylation between treatments.

## Methods

### Overview

To understand the functional role that *Dnmt1* plays during gamete formation of male *O. faciatus*, we used RNAi to knockdown *Dnmt1* gene expression at two sampling points post-treatment: 7 days and 14 days. After the treatment at two sampling points, we used confocal microscopy to phenotype the testes and determine the state of spermatocytes. We then profiled gene expression using RNA-seq of testes at the same two sampling points to understand how *Dnmt1* knockdown perturbed the testes transcriptome.

### Animal colony and husbandry

*Oncopeltus fasciatus* colony stocks were purchased from Carolina Biologicals (Burlington, NC). The colonies were maintained in Percival incubators under a 16:8 h light:dark cycle at 26 °C. The animals were fed organic raw sunflower seeds and had ad libitum deionized water. We needed to collect animals of known age so we removed eggs from the colonies and housed them in plastic storage containers with food and water. Nymphs were sexed at the fourth instar and separated into single sex colonies. Containers were checked daily for adult eclosions. New adults were placed into individual petri dishes with food and water.

### RNA interference (RNAi) synthesis, administration, and quality control

We created RNAi constructs of *Dnmt1* following Bewick et al. [8]. Briefly, DNA templates of *Dnmt1* were prepared via PCR. Following that, double stranded RNA was synthesized and then digested with an Ambion MEGAscript kit (ThermoFisher Sci, Waltham, MA) following the manufacturer’s protocol. This reaction was purified with a phenol:chloroform:IAA extraction followed by a sodium acetate precipitation. Concentration was measure by a Qubit using the ssRNA kit following the manufacturer’s protocol. Sense and anti-sense strands were then allowed to anneal. We used *eGFP* as an exogenous control construct. Before administration, the RNAi construct concentration was adjusted to 4 μg/μL in injection buffer (5 mM KCl, 0.1 mM NaH2PO4) for both *Dnmt1* and *eGFP*. Sexually mature, virgin males (~ 7-day post-adult ecolsion) were injected with 3 µL ds-*Dnmt1* RNA or *eGFP* using a pulled capillary needle between the third and fourth abdominal segments [10]. Males were paired with a virgin female to allow for mating and stimulate sperm production. Pairs were kept in petri dishes under standard rearing conditions until they sampled. Males were haphazardly allocated to treatment group.

We have established previously there is no difference between controls using RNA constructs with no specificity to *O. fasciatus* genome sequence (e.g., *eGFP*) and buffer alone injections [8]. Therefore, we did not include a buffer alone control. Previously, we have targeted RNAi constructs to two different regions of *Dnmt1*; the cytosine-specific DNA methyltransferase replication foci domain (RFD) and the DNA methylase domain (AdoMet). These give rise to identical phenotypes, which suggests minimal off-target effects [8]. Thus, here, we only targeted against the RFD consensus domain.

### RNAi validation by quantitative RT-PCR (qRT-PCR)

We assessed the effectiveness of *Dnmt1* knockdown, using qRT-PCR. We synthesized cDNA using 500 ng RNA with qScript cDNA SuperMix (Quanta Biosciences, Gaithersburg, MD). Validated primers were used from Bewick et al. [8]. GAPDH was used as the endogenous reference gene. We used Roche LightCycler 480 SYBR Green Master Mix with a Roche LightCycler 480 (Roche Applied Science, Indianapolis, IN) with 3 technical replicates of 10 μL reactions as previously reported [11]. We used the ΔΔ*C*_T_ method to estimate differences of expression using *eGFP* samples as our comparison group [29]. We had 14 7-day *eGFP*, 14 7-day *Dnmt1*, 14 14-day *eGFP*, and 13 14-day *Dnmt1* biological replicates.

### Phenotypic analysis of *Dnmt1* knockdown

Virgin, adult males were collected on the day of emergence. Males were injected with ds-*eGFP* or ds-*Dnmt1* as described above at 7–10-day post-adult emergence. Males were haphazardly assigned to two dissection days. One group of males were dissected 7-day post-RNAi treatment and a second group was dissected 14-day post-RNAi treatment.

Testes were dissected from the males in PBS and processed for microscopy as described below. To assess the activity of early stage spermatocytes, the testis tubules were stained with anti-phosphohistone H3 Ser 10 antibody (pHH3; Millipore antibody 06-570, Sigma Aldrich, St. Louis, MO). Anti-pHH3 stains for chromosome condensation in preparation for mitosis and meiosis [19, 38]. Primary antibody staining was visualized with a secondary antibody, Alexa Fluor goat–anti-rabbit 647 (ThermoFisher Scientific, Waltham, MA). Testis tubules were counterstained with DAPI (0.5 μg/mL PBT) to visualize nuclei. Negative controls in which primary antibody was absent showed no non-specific binding of the secondary antibody (data not shown.) Testis tubules were visualized on a Zeiss 710 confocal microscope or an EVOS (ThermoFisher Scientific, Waltham, MA).

To quantify the number of actively dividing spermatocysts, testis tubules from males were examined. Anti-pHH3 antibody stains both spermatogonia undergoing mitotic divisions and spermatocysts undergoing meiotic divisions (Fig. 1A). The numbers of positively stained spermatocysts in either control (ds-*eGFP*) or knockdown (ds-*Dnmt1*) males were compared at within each sample using a *t* test with unequal variance and the prediction that ds-*Dnmt1* samples would have fewer dividing cells (*n* = 20 per treatment, except 7-day *Dnmt1* had 18).

### RNA extraction

The testes of males of each treatment were dissected out in ice-cold 1X PBS at the appropriate sampling point, flash frozen with liquid nitrogen, and stored at − 80 °C until nucleotide extraction. Total RNA was extracted using a Qiagen Allprep DNA/RNA Mini Kit (Qiagen, Venlo, The Netherlands) following the manufacturer’s protocol. Homogenization of the testes were performed with a handheld Kimble pellet pestle in RLT buffer. Quantification was done with a Qubit fluorometer using the RNA BR kit.

### RNA-seq high-throughput library preparation and sequencing

The extracted RNA of *O. fasciatus* ds-*Dnmt1* and control (ds-*eGFP*) biological replicates at 7- and 14-day post-injection was used to construct poly-A selected Illumina TruSeq Stranded RNA LT Kit (Illumina, San Diego, CA) following the manufacturer’s instructions with limited modifications. The starting quantity of total RNA was adjusted to 1.3 µg, and all reagent volumes were reduced to a third of the described quantity. We targeted 10 M 2 × 150 bp read pairs per biological replicate using an Illumina NextSeq 500 with v3.1 chemistry. There were 13 7-day *eGFP*, 11 7-day *Dnmt1*, 16 14-day *eGFP*, and 15 14-day *Dnmt1* libraries. Libraries were sequenced at the Georgia Genomics and Bioinformatics Core (Athens, GA, USA).

### Read quality control and mapping

Reads were initially assessed for quality with fastQC (v0.11.9; default settings; [5] to establish a baseline. Reads had adapters trimmed with cutadapt (v2.8,–trim-n -O 3 -u -2 -U -2 -q 10,10 -m 30; [33] using the TruSeq adapter sequences. Reads were again assessed with fastQC with default settings. Overlapping reads were combined with FLASh (v 1.2.11, default settings; [31]. As a final QC step, reads that mapped to rRNA genes were removed with SortMeRNA (v4.3.3, all gff entries annotated as rRNA) [25].

We used the NAL i5k *O. faciatus* genome (the “BCM-After-Atlas” version) and annotation (Official Gene Set v1.2; [35]. These were downloaded from the NAL i5k site: https://i5k.nal.usda.gov/content/data-downloads. This was the most current version of the genome and gene annotation at the time of analysis. HISAT2 (v2.2.1; no soft-clipping; [24] was used to map reads to the genome (Additional file 1. Extended reads (i.e., reads that were combined by FLASh had ~ 30% higher mapping rate. Mappings were converted to read counts by StringTie (v2.1.7; [37] following the manual instructions for export to DESeq2.

### Functional annotation

We updated the functional annotation of the *O. faciatus* proteome/transcriptome using eggNOG-mapper webserver at the level of Insecta (http://eggnog-mapper.embl.de/; v 2.19; [9]. This annotated 12,526 of 19,615 gene models (63.8%) with Gene Ontology terms, which allowed us to perform a GO term enrichment analysis and a GO term overrepresentation analysis.

### Differential gene expression

Read counts were imported into R using tximport (Bioconductor v1.20.0; [44] following the manual’s instructions. We used R (v4.1.0 [40], within an RStudio IDE (build 492 [41], for the differential gene expression analysis.

DESeq2 (Bioconductor v1.32.0; default settings; [30] was used for all DGE analyses following the programmers’ suggestions for exploratory data analysis and sample/analysis quality control. *eGFP* was set and used as the comparison group for all analyses. 7-day and 14-day samples were analyzed separately as previously discussed to generate a list of differentially expressed genes between control and experimental treatments. The model matrix specification was also checked to ensure correct specification (i.e., that program was contrasting the samples in the correct way). After importing and initial analysis, samples were plotted with a PCA to visually check for outliers according to the manual’s recommendation. One 7-day *Dnmt1* sample was removed. Two 14-day samples were also removed—one *Dnmt1* and one *eGFP*. All were removed due to poor library quality.

After removal of the outliers each analysis was repeated using the same settings of DESeq2 and the results were again quality controlled. We used the default dispersion estimator and shrinkage method, apeglm [50]. We used *s* values to estimate statistical significance after false discovery rate correction at the level of 0.05 [45]. Results were visualized with the fviz_pca_ind function of the factoextra R package [23].

We also compared the overlap of the differentially expressed genes here to the differentially expressed genes of *O. fasciatus* ovaries under the same treatment scheme using the intersect function of R. A simulation analysis was conducted to see if any overlap observed was statistically significantly enriched or depleted [12].

### a priori Candidate gene screen

The first test of our hypothesis was to assess the influence *Dnmt1* knockdown had on a series of candidate genes. These genes were chosen based on literature searches for genes involved in meiosis and spermatogenesis of insects. These include cell fate and proliferation genes that we did not expect to be differentially expressed (members of the *Wnt* and *Frizzled* families), cell cycle control genes some of which we did expect to be differentially expressed (*Vasa*) and some of which did not expect to be differentially expressed (*Cyclin B3*, *Cyclin D2*, *Cyclin D3*, *Cell Division Cycle 20*, *Cell Division Cycle 25* family members), maintenance of chromosome genes that we did expect to be differentially expressed (*Structural Maintenance of Chromosome 3* family members), meiotic transition gene that we did expect to be differentially expressed (*Boule*), and meiotic recombination genes that we did expect to be differentially expressed (*SPO11 Initiator of Meiotic Double Stranded Breaks*, *MutS Homolog 5*, *Meiotic Nuclear Divisions 1*, *Homologous-Pairing Protein 2*). None of the candidates had specific directional changes, except decreased expression of *Vasa* and *Boule* within the *Dnmt1* knockdowns. We extracted the raw P values for expression differences from the DESeq2 results and compared them with FDR corrected P values we generated using the Benjamini–Hockberg procedure [6].

### a priori GO term enrichment analysis

As secondary test of our hypothesis, we selected five high-level GO terms that represented the cellular processes that we thought were being perturbed or not after *Dnmt1* knockdown. These were GO:0007049 Cell Cycle, GO:0000278 Mitotic Cell Cycle, GO:0051321 Meiotic Cell Cycle, GO:0007276 Gametogenesis, and GO:0007283 Spermatogenesis. With our previous observed phenotypes and the one observed with the microscopy here, we expect Meiotic Cell Cycle, Gametogenesis, and Spermatogenesis to be enriched. We expected Cell Cycle and Mitotic Cell Cycle not to show enrichment. We included GO:0070265 Necrotic Cell Death and GO:0006915 Apoptotic Process to assess if cells were undergoing these processes or if they had simply arrested. We used S values from DESeq2 to rank order all expressed genes at 7 and 14 days separately. We used fgsea R package [26] with default parameters, except allowing 1,500 genes as a maximum, to test if genes annotated with these GO terms were enriched at the top of these lists. Results were visualized with the same R package.

### Gene ontology (GO) term overrepresentation analysis

We found overrepresented terms to give us biological processes that might be perturbed using the topGO package of R (v2.48.0; [2]. This analysis is often termed an enrichment analysis [21], but statistically it tests if there is an overrepresentation of a GO term from a list of interesting genes (usually those that are differentially expressed compared with the expectation of the same number of random picks. It does not calculate if a specific GO term (or another type of annotation, e.g., KEGG is enriched at the beginning of an ordered gene set. We performed GO term tests using Fisher’s exact test with the weighted algorithm. GO terms of genes of interest were compared to all expressed genes within our samples. Updated GO terms can be found in Additional file 2.

We performed a semantic similarity analysis using REVIGO using default settings [46] to refine our overrepresented GO terms to even higher level summaries of the processes involved and identify central processes enriched from our treatments.

## Supplementary Information

**Additional file 1. **Contains the metadata for the RNA-seq libraries used here.

**Additional file 2. ** Contains the updated GO term annotations for *Oncopeltus fasciatus*.

**Additional file 3. ** Contains the output from DESeq2 for the differential gene expression of the 7-day samples.

**Additional file 4. ** Contains the output from DESeq2 for the differential gene expression of the 14-day samples.

**Additional file 5. **Contains the output from topGO for the GO term overrepresentation analysis of the 7-day samples.

**Additional file 6. **Contains the output from topGO for the GO term overrepresentation analysis of the 14-day samples.

**Additional file 7. **Contains the output from topGO for the GO term overrepresentation analysis of the Bewick et al. [9] and 14-day samples overlap

**Additional file 8. **Contains the raw data for figure one, nuclei count of dividing gametes.

## Data Availability

All high-throughput data are available under NCBI BioProject # PRJNA957711. Data for testes tube nuclei are provided in Additional file 8.
